# Tourists’ apprehension toward choosing the next destination: A study based on the learning zone model

**DOI:** 10.3389/fpsyg.2022.987154

**Published:** 2022-08-25

**Authors:** Adriana Manolicǎ, Diana-Sînziana Ionesi, Lorin-Mircea Drǎgan, Teodora Roman, Patricia Elena Bertea, Gabriela Boldureanu

**Affiliations:** Department of Management, Marketing and Business Administration, Faculty of Economics and Business Administration, Alexandru Ioan Cuza University of Iaşi, Iaşi, Romania

**Keywords:** learning zone model, comfort zone, fear zone, learning zone, growth zone, tourist destination

## Abstract

The current research is based on Senninger’s Learning Zone Model applied to the tourists’ comfort zone. This model was created in 2000 and it proved to be useful in many applied areas: Psychology, Sociology, Marketing and Management. This modes is a behavioral one and shows how a person can justify his action based on previous tested experiences (comfort zone) or dares to step beyond in fear, learn or growth zone. Our research is extending the existent area of expertise to tourism. We aimed at exploring whether the tourists’ apprehension toward choosing their next destination from a comfort zone perspective or rather from the other zones’ perspectives such as fear, learning or growth. To meet this purpose we conducted a mixed method: firstly a qualitative one, an in-depth interview based on Delphi method with 10 tourism specialists and secondly an online survey on 208 Generation Z tourists. The interviews were meant to help developing a 20 items scale (5 items for each level of the model) to measure from which of the 4 zones are the respondents making the choice of the future travel destination. Our conclusions show that Gen Z tourists display behaviors that can be associated with learning or growth zones rather than the comfort zone. This is relevant when choosing the next travel destination, because our findings could bring about a new approach to promoting tourist destinations as part of various products. As a result, a large range of managerial tools can better adapt the promotion messages to the target market from a new psychological perspective.

## Introduction

The comfort zone could be associated with a warm and familiar hug, nevertheless, psychologists consider it beneficial and restrictive at the same time (McWha et al., 2018). This field of research has been popular with a variety of specialists such as mental health practitioners, behavior therapists, and other psychologists (Gilligan and Dilts, 2009). The paradox noticed by many is that while finding oneself in the comfort zone provides calm and quietness (Passafaro et al., 2021), at the same time it might prevent growth (Santoro and Major, 2012; Woodward and Kliestik, 2021). The solution researchers seem to agree upon is to balance those two divergent forces (the one that keeps us wanting to remain still and the one that makes us wanting to grow) to improve our lives (Berno and Ward, 2005).

Bardwick (1995) coined the term “comfort zone” in the management context, in order to help assess more efficiently the motivation behind certain employees’ behaviors. Inside of the comfort zone, the stimulus for performance growth seems scarce. While the routine generally averts risks it can also limit human resources development. That is why Karwowski (2018) considered that this concept also applies to the field of behavioral psychology.

Our comfort zone is considered to be a psychological, emotional, and behavioral construct (Lichy and Favre, 2018; Nica et al., 2022a) that defines our daily routine and involves familiarity, safety, and security. Although we often hear professors, coaches, or motivational speakers encouraging us to reach beyond our limits and explore activities outside our regular boundaries, this ignores a fundamental reality, namely the existence of personal differences among individuals. Someone’s comfort zone might be completely different from another’s.

Each person has his/her comfort zone modeled by herself, a healthy adaptation to achieve an emotional balance free from anxiety. It is a place where a person feels calm, comfortable, and relaxed. However, experimenting with a reasonable amount of stress or anxiety from time to time can prove beneficial. Miller (2019) refers to the comfort zone as an illusion, a self-imposed mental limitation that is not easy to overcome. The difficulty of overcoming this limitation is mostly linked to the fear of missing the warmth and calm of our imaginary cocoon (Nica et al., 2022b).

Page (2020) summarized the most relevant 4 benefits of moving beyond this comfort zone: (i) self-fulfillment, a term retrieved from the classical hierarchy of needs formulated by Maslow (1943) and (ii) growth mindset, a term defined by Dweck (2000) as relying on flexibility, trial and error and unlimited potential as opposed to a fixed mindset where people believe there is a personal threshold for everyone beyond which advance become problematic. (iii) antifragility which regard volatility, hazard, chaos, and stress as push factors for self-development and prosperity (Taleb, 2014), and (iv) self-efficacy explained by Bandura (1997) as the sum of actions to be executed to reach a certain objective.

An interesting approach developed by Senninger (2000) is the Learning Zone Model. According to this model, the fear which settles in once the comfort zone is left behind does not necessarily indicate reaching the panic zone. It is more of a natural emotion accompanying moving into the learning and growth zones.

Once all these obvious advantages of stepping out of the comfort zone are taken into account, the essential question is to find out from which one of the four zones (comfort/fear/learning or growth) is the Gen Z consumer reacting when choosing the next tourist destination?

We want in this paper to investigate, starting from Senninger’s model, Generation Z travelers, aged 18–27, in order to discover which one/ones out of the four zones (comfort/fear/learning or growth) is the most important for them when it comes to choose the next travel destination. For that reason we conducted a mixed method research. Firstly, with the help of tourism and travel specialists (Delphi method), we created a 20 items questionnaire (5 for each zone) and secondly we applied it in an online survey on 208 Gen Z individuals. We set as a research objective the identification of certain behavioral patterns of Gen Z consumers who are currently in their comfort zone.

Section 2 of this paper presents the details of our research design and the following parts describe the findings, discussions and conclusions. This would serve future research on solutions and actions for taking them to the superior level of this model, namely the growth zone, overcoming feelings of fear and anxiety which prevents this progress.

## Literature review

### The learning zone model

This model was developed initially by Vygotsky (1978), later on, the definitive version belonged to Senninger (2000). The underlying idea is that in order to learn and progress we need to be challenged and stimulated (Kliestik et al., 2022). It is all about the balance of forces. If we are not pushed enough, the probability that we move beyond the comfort zone is rather low, while if we are pushed too hard, the risk is to panic and feel overwhelmed. Both situations lack a proper balance and entail limited learning (Senninger, 2000).

The model has two variations: a limited one with only three zones and an expanded one with four zones. We based our research on the latter. The comfort zone provides a familiar and safe feeling and entitles the subjects of it to feel in control. It is a risk-free area that is also not very eventful (Karwowski, 2018; Kovacova et al., 2022). A state of reaching a plateau besides monotony and boredom settles in Kovacova et al. (2022). Often, people tend to conform to it and even put the effort into maintaining it (Kliestik et al., 2022). However, as life moves on, a series of internal and external factors trigger changes (Dweck, 2005; Kliestik et al., 2022). We might get sick, change our job or our family might expand and all these push us outside of our comfort zone.

As soon as we move out of our comfort zone we find ourselves in the fear zone. There, a process of self-inquiry about our choices might occur. It is possible that we face a low self-confidence situation and doubt settles in Miller (2019). Sometimes we internalize critical voices which have a paralyzing effect on ourselves (Senninger, 2017). Often, we can be scared to the point when we regret moving out of our comfort zone and rush back inside of it (Andronie et al., 2021). Meanwhile, we might start complaining more and focus on obstacles and issues to justify this embarrassing return (Wallace and Lãzãroiu, 2021).

Once we get close enough to the learning zone we score the first victory: we passed the fear zone and we suppressed the internal and external critical voices (Page, 2020). In the learning zone we face new challenges, but we tend to prioritize solutions over problems (Lyons, 2022). In other words, we move from a pessimistic to an optimistic perspective and this allows us to grow (Pearce and Packer, 2013).

The growth zone might be equated with the terminus point for this psychological pursuit. Here, the old fears are slowly receding even if new ones might settle in. The advantage is that we became more resilient during this phase and we learned to set more ambitious goals for ourselves (Lǎzãroiu and Harrison, 2021). As long as our personal development continues our lives gather more sense. Progressively, we define superior objectives, and we create a long-term-based personal view (Pongelli et al., 2021).

This model, besides its significant contribution to human psychology development, remains a resource with robust applied configuration (Dweck, 2005; Kliestik et al., 2022). Our work intends to explore how this model could be applied to understanding tourists’ behaviors.

Intentionally leaving the comfort zone can be possible only by developing a growth mindset. While a rigid mindset keeps us in the prison of the fear of failure, a growth mindset expands opportunities and possibilities. It inspires us to overcome fear, to take healthy risks, to learn new lessons and the outcomes are blooming in all life dimensions (Perruci and Warty Hall, 2018).

When it comes to learning, Elbæk et al. (2022) are presenting the effects of Yerkes-Dodson law that stipulates that there is an empirical relationship between stress and performance. In other words, that there is an optimal level of stress that corresponds to an optimal level of performance. Based on Yerkes-Dodson law, learning is possible not only beyond comfort zone, but also beyond fear. Is not defined by stress. Quite the opposite! It is a space for opportunities, where, in order to optimize the performance, people must reach a certain level of stress, higher than normal. So we obtain what they call to be an optimal anxiety.

Comfort zone proves to be nothing but a cozy place to live in, and its only reason is to prepare you for all the challenges in life (Anichiti et al., 2021). Anxiety, fear and stress improve performance until a certain level—called optimum stimulation level. Beyond this point, performance drops while stress is increasing (Avornyo et al., 2019).

What we can see is that comfort, fear and learning are strongly related (Perruci and Warty Hall, 2018). Learning zone model developed by Senninger can be justified by seeking balance (Freeth and Caniglia, 2020). We must exit our comfort zone long enough to reach optimal anxiety, but not too much, for not letting anxiety to take control.

Moreover, all our decisions are facing these mirrors: the comfort mirror—showing the future self that keeps our *status quo*; the fear-mirror—presenting the possible panic we have to face in near future; the learning-mirror—with all the lessons we have the assimilate and the growth-mirror—that is indicating the future self we want to become. And, by analyzing all these projections, our mind is developing a cost-benefit analysis (Zheng et al., 2021). As long as we stay in our comfort zone, the benefits are small but guaranteed. We feel good, safe and we are not in danger. However, if we don’t change a thing, we cannot expect something spectacular to happen. If we remain there for a long time, we can limit ourselves, sinking into boredom and monotony.

Plog (1974), examined the motivations of travelers and arrived at the classification of tourists starting from two approaches: allocentric and psychocentric. Allocentric tourists, or often called ‘wanderers‘, are brave enough to travel to the unknown. They like adventure and would not mind if they were the first to explore a certain area. Allocentric tourists will often travel alone, without the need for a guide. They enjoy cultural tourism, are ethical travelers and love to learn. Stainton (2022) suggested that only 4% of the population is expected to be purely allocentric, most are on Plog’s scale in the category of close or centric cluster. Allocentric tourists have some common features: they are independent travelers, they like adventure; they are eager to learn and like to experience unfamiliar things; they are not followers of mass tourism, tourist packages and group excursions; they are fans of cultural tourism, being ethical tourists; love challenges; prefers sustainable tourism and slow tourism (as opposed to mass tourism). All this being said, making an analogy with the characterization of the four areas of Senninger (2000), Learning Model allocentric tourists are rather those who are in the growth zone or in transition from the learning zone to the growth zone.

At the opposite side are psychocentric or ‘repeating‘ tourists. They are most often associated with well-developed or overdeveloped areas for tourism. They will choose holiday destinations that have already been “tested,” where they can feel comfortable and familiar. The portrait of a psychocentric tourist (Stainton, 2022), looks like this: he/she enjoys familiarity and likes the chosen destination to offer him/her the comfort of home; prefers well-known brands; often travels in organized groups; is a supporter of holiday packages and all-inclusive holidays; spends a lot of time in the holiday resort and doesn’t know much about the local culture; he/she is not open to learning new things about the area he visits or about the people who live there; pays a single flat fee to cover most of the holiday costs and is a regular visitor to the same resort/destination. This typology, without a doubt, can be associated with the comfort zone, being mentioning key words such as: “comfortable,” “familiar,” “known,” “regular,” “organized” etc.

The reality is that not many tourists fit perfectly into the two typologies at the extremes (Stainton, 2022), respectively, allocentric and psychocentric. And this is why Plog has developed a scale, through which tourists can be placed anywhere along the spectrum. So, the largest category of tourists falls somewhere in the mid-centered category of the spectrum. Mid-center tourists like to have a little adventure, but also something from the comfort of home. Maybe they book their vacation by means of an interesting announcement, but then they spend most of their time in the holiday resort. Or maybe they choose an organized trip, but then they choose to break away from the crowd and explore the local area (Stainton, 2022). These tourists are best suited to the fear zone, where there is a battle between staying in the comfort zone and progressing further toward the learning zone.

Plog (1974) created a fundamental model in travel and tourism research. His theory has encouraged critical thinking throughout the tourism community for several decades. Our paper goes beyond Plog’s model, being enriched by Senninger (2000) explanations of consumer psychology in the face of a purchasing decision. We aim to explore these types of tourists from the perspective of the learning area from which they chose to make the travel decision.

## Methodological approach

### Research context

The current research explores the psychographic and behavioral factors determining the choice of a certain tourist destination. It was targeted at the Generation Z adult population within the age range 18–27. The research is based on the Learning Zone Model formulated by Senninger (2000) which features four zones: comfort, fear, learning, and growth. The research results, conclusions, and suggestions will constitute a reference point for formulating various marketing strategies. Those marketing strategies include promoting a tourist destination once the profile of Gen Z tourist is defined according to the 4 above-mentioned zones. Therefore, personalized marketing messages can emerge aiming at for example diminishing the fears and uncertainty of those in the comfort or fear zones or attracting those in the learning or growth zone through new experiences, adventure, and other challenges. Each destination has one or more target markets and a tourist typology-based learning zones model might be a relevant variable when segmenting the market for Gen Z tourists.

### Research design

The purchase decisions of Gen Z tourists are largely emotional and can be attributed to certain zones of the learning zone model developed by Senninger (2000). The consumer acts from within a certain zone such as comfort, fear, learning, or growth. This can lead to certain behavioral patterns when choosing a tourist destination. We devised the following research question:

From which one of the four zones (comfort/fear/learning or growth) is the Gen Z consumer reacting when choosing the next tourist destination?

The research had 2 main phases

Phase 1 involved qualitative research aimed at identifying the keywords corresponding to each of the 4 zones part of the model (comfort, fear, learning, and growth). We planned to do this by exploring tourism specialists’ views. The resulting keywords were subsequently integrated into the quantitative research instrument. The objectives set for phase 1 were:

O1.1: Generating keywords for describing the behavior of tourists who are in the comfort zone as per the Learning zone model (Senninger, 2000).
O1.2: Generating keywords for describing the behavior of tourists who are in the fear zone as per Learning zone model (Senninger, 2000).
O1.3: Generating key words for describing the behavior of tourists who are in the learning zone as per Learning zone model (Senninger, 2000).
O1.4: Generating key words for describing the behavior of tourists who are in the growth zone as per Learning zone model (Senninger, 2000).

Phase 2 consisted of quantitative research directed toward analyzing the Gen Z tourists’ perspectives from Iasi, Romania. Their perspective was scrutinized corresponding to the Learning Zone Model (comfort, fear, learning, and growth zones) in terms of choice of their future travel destination. More precisely we focused on learning from which of the 4 zones are they making this choice. The objectives set for phase 2 were

O2.1: Identifying the Gen Z tourist profile (among those living in Iasi). Profiling is based on tourism services purchase frequency, distance traveled, domestic/outbound destinations preference, type of holiday, travel motivation, and travel budget.
O2.2: Identifying specific behavior related to their comfort zone for Gen Z tourists from Iasi as far as their next holiday choice is concerned.
O2.3: Identifying specific behavior related to their fear zone for Gen Z tourists from Iasi as far as their next holiday choice is concerned.
O2.4: Identifying specific behavior related to their learning zone for Gen Z tourists from Iasi as far as their next holiday choice is concerned.
O2.5: Identifying specific behavior related to their growth zone for Gen Z tourists from Iasi as far as their next holiday choice is concerned.

### Research hypotheses

For phase 2 of the research (online survey), we formulated the four hypotheses.

As Stainton (2022) stated, based on Plog (1974) model, there are psychocentric or ‘repeating‘ tourists. Our sample of experts have characterized them with words like: comfort seekers, valuing control and security, having a strong aversion against risk and willing to repeat positive experiences. So, after Plog (1974); Stainton (2022) and justified by the choices made by our group of experts, we can formulate the first hypothesis:

H1: There is a connection among the attributes of the comfort zone as per The Learning Model Zone (Senninger, 2000) corresponding to choosing of a tourist destination.

Elbæk et al. (2022) argued that there is a relationship between stress and performance. They said that it is necessary to step into fear in order to thrive. Fear and stress can be challenging, but only until a certain point, beyond what performance is not possible. When it comes to analyze tourists behavior facing fear, our group of specialists selected words like: being suggestible, overcoming fear of unknown and challenges, willing to experience and being courageous. Based on that we formulated the second hypothesis:

H2: There is a connection among the attributes of the fear zone as per The Learning Model Zone (Senninger, 2000) corresponding to choosing of a tourist destination.

Comfort, fear and learning are strongly related (Perruci and Warty Hall, 2018). We must exit our comfort zone long enough to reach optimal anxiety, but not too much, for not letting anxiety to take control (Freeth and Caniglia, 2020). Only those who are willing to learn and keep an open mind are thriving (Anichiti et al., 2021). That is why our group of specialists selected the following words to describe a person that makes a decision justified by his/hers learning zone: is open to novelty, curious, interested in learning new things, loves challenges and risk taking, being explorer and adventurous. As a consequence, the third hypothesis is:

H3: There is a connection among the attributes of the learning zone as per The Learning Model Zone (Senninger, 2000) corresponding to choosing of a tourist destination.

Leaving the comfort zone can be possible only by developing a growth mindset (Perruci and Warty Hall, 2018). Our mind is developing a cost-benefit analysis (Zheng et al., 2021) and puts into balance the cost of leaving the comfort zone with the benefit of reaching the growth zone. Those who see mainly the benefits are, according to our group of specialists: decisive, emotionally developed, willing to fulfill ideals and objectives, committed to their personal growth and seeing traveling as a lifestyle. These conclusions helped us formulate the fourth hypothesis:

H4: There is a connection among the attributes of the growth zone as per The Learning Model Zone (Senninger, 2000) corresponding to choosing of a tourist destination.

### Research methods

Phase 1: Semi-Structured interview applied to tourism specialists using the Delphi method. There were 10 experts in tourism (tourism agents, bloggers, and tourism master graduates).

Phase 2: Quantitative research based on an online survey having 208 respondents among travel enthusiastic from Iasi. The gender split was 87 and 121 female respondents, aged 18–27, corresponding to Gen Z.

### The research instruments

Phase 1: We used a selection questionnaire for selecting the participants. The interviews required answers regarding the profiling of a tourist who makes the purchase decision from his comfort, fear, learning, or growth zone.

Phase 2: Based on the specialists’ answers, the questionnaire items were realized. The instrument had 3 sections:

Section 1 was built around determining the profile of Gen Z tourists and consisted of 8 questions. The questions asked the participants to associate the travel with a random word, to assess their travel frequency, preference for either domestic or international destinations, the maximum distance they were eager to travel, holiday type and motivation, and their weekly travel budget.
Section 2 consisted of 4 sets of 5 statements each using keywords defining tourists from each of the 4 zones (comfort, fear, learning, growth). The statements were based on the specialists’ answers collected through the semi-structured interview described earlier. Respondents were asked to grade the statements on a scale from 1 to 10 where 1 meant full disagreement and 10 full agreement. Each construct contains a so-called key-statement which is formulated based on the most representative key-word.

**Comfort zone:**
  I want to feel in control.
  I choose on safety criteria (personal safety, transport, destination, etc.).
  I try to reduce the risk of unforeseen events, which could take me out of my comfort zone.*
  I prefer to repeat positive experiences I have had in the past.
  I avoid any complications that may occur.

**Fear zone:**
  I always want to gather new experiences.
  I try to overcome my fear of the unknown.*
  I let myself be influenced by the opinions of those around me.
  I leave room for the unexpected.
  I accept new challenges, giving up excessive planning.

**Learning zone:**
  I am open to all experiences.
  I allow myself to always be curious.
  I leave room for adventure.
  I am willing to learn new things.*
  I love challenges.

**Growth zone:**
  I am always determined on what I want.
  I consider any experience that contributes to my personal growth.*
  I am getting closer to fulfilling my dreams as a tourist.
  I am looking for experiences that will enrich my soul.
  I consider traveling as a lifestyle.

Section 3 consisted of social and demographic questions for identifying the respondents.

## Research results

### Phase 1

For this initial phase of our research, namely the semi-structured interview using the Delphi method, we inquired a group of 10 tourism experts from Iasi. The objective was to identify keywords in defining the tourists choosing travel destinations from one of the 4 zones of The Learning Zones Model of Senninger (2000). Table 1 shows the prevalence of the most frequently mentioned keywords.

**TABLE 1:** The prevalence of the most frequently used keywords or expressions describing a tourist according to Senninger’s model.

“Comfort zone”		“Fear zone”		“Learning zone”		“Growth zone”	
*Keyword/Expression*	*Frequency*	*Keyword/Expression*	*Frequency*	*Keyword/Expression*	*Frequency*	*Keyword/Expression*	*Frequency*
Comfort	**4**	Suggestible	**4**	Openness to novelty	**4**	Decisive	**5**
Control	**4**	Overcoming fear of unknown	**3**	Curious	**3**	Fulfilling objectives, ideals	**4**
Risk aversion	**3**	Inclination to experiment	**3**	Interest in learning new things	**3**	Emotional development	**3**
Repeating positive experiences	**3**	New challenges	**3**	Loves challenges	**2**	Personal growth	**3**
Security	**3**	Courageous	**2**	Risk taking	**2**	Travelling as lifestyle	**3**
Comfort seeker	**2**	Curious	**2**	Explorer	**2**	Risk taking	**2**
Lack of complications	**2**	Doubtful	**2**	Adventurous	**2**	Passionate	**2**
Uncertainty avoidance	**2**	Lack of excessive planning	**2**	Determined	**1**	Enthusiast	**1**
Monotony	**2**	Phobia	**1**	Development	**1**	Extrovert	**1**
Anticipation	**1**	Paranoid	**1**	Motivation	**1**	Progressive	**1**
Conforming	**1**	Stress	**1**			Opened	**1**

Source: own computation.

The keywords and expressions provided by the 10 tourism specialists were centralized as per Table 1. We, therefore, achieved all 4 objectives and ranked the keywords and expressions according to their prevalence. For the comfort zone, the following keywords or expressions were selected: comfort, security, risk aversion, repeating positive experiences, and control. For the fear zone, the following keywords or expressions were selected: suggestible, inclination to experiment, overcoming fear of the unknown, new challenges, and lack of excessive planning. For the learning zone, the following keywords or expressions were selected: openness to novelty, curious, Interest in learning new things, loves challenges and adventurous. For the growth zone, the following keywords or expressions were selected: decisive, fulfilling objectives and ideas, personal growth and emotional development.

### Phase 2

We analyzed the results of the survey for each stated objective.

O2.1: Identifying the Gen Z tourist profile (among those living in Iasi). Profiling is based on tourism services purchase frequency, distance traveled, domestic/outbound destinations preference, type of holiday, travel motivation, and travel budget.

We exported the data from the SPSS software using the “Descriptive Statistics” function in order to obtain the prevalence. According to the Dimensional Analysis, we created the profile of a Gen Z tourist from Iasi. S/he associates travel mostly with relaxation, freedom feelings, adventure, and experience. S/he travels on average 6 times a year and prefers equally domestic and international destinations. We noted an inclination to travel to a maximum distance of 2,300 kilometers from home and he enjoys mostly 2 types of holidays: resort holidays and city breaks. Whenever s/he chooses a holiday destination a number of attributes are sought: relaxation, having fun, exploring nature, understanding local history, and culture, and adventure. The Gen Z tourist from Iasi allocates on average a weekly travel budget of approximately 2,400 RON (500 euros).

O2: Identifying specific behavior related to their comfort zone for Gen Z tourists from Iasi as far as their next holiday choice is concerned.

H1: There is a connection among the attributes of the comfort zone as per The Learning Model Zone (Senninger, 2000) corresponding to choosing of a tourist destination.

For verifying this hypothesis we performed a correlation test for the variable attributes of the comfort zone based on r Pearson correlation. We used SPSS software for this end.

According to Table 2, each correlation significant because Sig is 0.000 (< 0.05) and it consists of a direct correlation (*r* > 0). The differences are based on the strength of the correlation between 2 variables. The strongest correlation within the comfort zone is between the risk and uncertainty avoidance, the r-value being 0.699 which indicated a strong correlation. Another strong correlation was found between “security inspired choices” and “risk avoidance” with an r-value of 0.654 although this correlation does not involve causality. Among “security inspired choices” and “uncertainty avoidance” we found an average to good correlation, Pearson r-correlation being 0.523.

**TABLE 2:** Pearson correlation for the comfort zone variables.

		Inclination to be in control	Security inspired choices	Risk avoidance	Positive experiences repeat	Uncertainty avoidance
Inclination to be in control	Pearson Correlation (r)	1				
	Sig. (2-tailed)					
Security inspired choices	Pearson Correlation (r)	0.446	1			
	Sig. (2-tailed)	0.000				
Risk avoidance	Pearson Correlation (r)	0.390	0.654	1		
	Sig. (2-tailed)	0.000	0.000			
Positive experiences repeat	Pearson Correlation (r)	0.266	0.403	0.376	1	
	Sig. (2-tailed)	0.000	0.000	0.000		
Uncertainty avoidance	Pearson Correlation (r)	0.292	0.523	0.699	0.364	1
	Sig. (2-tailed)	0.000	0.000	0.000	0.000	

Source: own computation.

We used Cronbach’s Alpha coefficient of reliability to identify the measure of internal consistency among the items defining the comfort zone, calculated by SPSS software. The aim was to check whether the items contribute to the comfort zone significance or not. The value of Cronbach’s Alpha was 0.779 which indicated a good consistency (Tavakol and Dennick, 2011). A side note would be that once the item “inclination to be in control” is removed the consistency improves. As a conclusion of this test, we can state that the comfort zone items do have an acceptable consistency which means there is consistency among the answers given by respondents for this dimension. This will lead to identifying the specific behaviors of tourists choosing a certain destination from their comfort zone.

O3: Identifying specific behavior related to their fear zone for Gen Z tourists from Iasi as far as their next holiday choice is concerned.

H2: There is a connection among the attributes of the fear zone as per The Learning Model Zone (Senninger, 2000) corresponding to choosing of a tourist destination.

We performed a Pearson correlation test to verify this hypothesis. We aimed at measuring the correlation among the variables defining the fear zone. This test was performed through SPSS software and the results are summarized in Table 3.

**TABLE 3:** Pearson correlation for the fear zone variables.

		Inclination to experiment	Overcoming fear of unknown	Suggestible	Unforeseen events	Lack of excessive planning and acceptance of new challenges
Inclination to experiment	Pearson Correlation (r)	1				
	Sig. (2-tailed)					
Overcoming fear of unknown	Pearson Correlation (r)	0.534	1			
	Sig. (2-tailed)	0.000				
Suggestible	Pearson Correlation (r)	0.156	0.207	1		
	Sig. (2-tailed)	0.024	0.003			
Unforeseen events	Pearson Correlation (r)	0.297	0.329	0.233	1	
	Sig. (2-tailed)	0.000	0.000	0.001		
Lack of excessive planning and acceptance of new challenges	Pearson Correlation (r)	0.467	0.454	0.215	0.627	1
	Sig. (2-tailed)	0.000	0.000	0.002	0.000	

Source: own computation.

As per Table 3, all correlations are positive for the fear zone, Pearson r correlation displaying beside the positive values significant correlation (Sig < 0.05). The strongest correlation within the fear zone is between “Lack of excessive planning and acceptance of new challenges” and “unforeseen events” with an r correlation value of 0.627 indicates an average to good correlation. A second moderate correlation can be noticed between “Inclination to experiment” and “overcoming fear of the unknown: (*r* = 0.534).

We used Cronbach’s Alpha coefficient of reliability to identify the measure of internal consistency among the items defining the comfort zone, calculated by SPSS software. The aim was to check whether the items contribute to the comfort zone significance or not. The value of Cronbach’s Alpha was 0.772 which indicated a good consistency (Tavakol and Dennick, 2011). This consistency could be improved if the item “suggestible” was eliminated.

As a conclusion of this test, we can state that the fear zone items do have an acceptable consistency which means there is consistency among the answers given by respondents for this dimension. This will lead to identifying the specific behaviors of tourists choosing a certain destination from the fear zone.

O4: Identifying specific behavior related to their learning zone for Gen Z tourists from Iasi as far as their next holiday choice is concerned.

H3: There is a connection among the attributes of the learning zone as per The Learning Model Zone (Senninger, 2000) corresponding to choosing a tourist destination.

We performed a Pearson correlation test to verify this hypothesis. We aimed at measuring the correlation among the variables defining the fear zone. This test was performed through SPSS software and the results are summarized in Table 4.

**TABLE 4:** Pearson correlation for the learning zone variables.

		Openness to novelty	Curiosity	Adventurous	Interest in learning new things	Loves challenges
Openness to novelty	Pearson Correlation (r)	1				
	Sig. (2-tailed)					
Curiosity	Pearson Correlation (r)	0.541	1			
	Sig. (2-tailed)	0.000				
Adventurous	Pearson Correlation (r)	0.781	0.545	1		
	Sig. (2-tailed)	0.000	0.000			
Interest in learning new things	Pearson Correlation (r)	0.588	0.547	0.672	1	
	Sig. (2-tailed)	0.000	0.000	0.001		
Loves challenges	Pearson Correlation (r)	0.691	0.503	0.742	0.601	1
	Sig. (2-tailed)	0.000	0.000	0.002	0.000	

Source: own computation.

All correlations presented in Table 4 are significant (Sig = 0.000 < 0.05) and we see positive correlation (r correlation > 0). In terms of their strength, we see within this dimension reasonable, good, or strong correlations.

We used Cronbach’s Alpha coefficient of reliability to identify the measure of internal consistency among the items defining the learning zone, calculated by SPSS software. The aim was to check whether the items contribute to the comfort zone significance or not. The value of Cronbach’s Alpha was 0.890 which indicated a good to strong consistency (Tavakol and Dennick, 2011). This consistency could be slightly improved if the item “curiosity” was eliminated.

As a conclusion of this test, we can state that the learning zone items do have an acceptable consistency which means there is consistency among the answers given by respondents for this dimension. This will lead to identifying the specific behaviors of tourists choosing a certain destination from the learning zone.

O5: Identifying specific behavior related to their growth zone for Gen Z tourists from Iasi as far as their next holiday choice is concerned.

H4: There is a connection among the attributes of the growth zone as per The Learning Model Zone (Senninger, 2000) corresponding to choosing of a tourist destination.

We performed a Pearson correlation test to verify this hypothesis. We aimed at measuring the correlation among the variables defining the fear zone. This test was performed through SPSS software and the results are summarized in Table 5:

**TABLE 5:** Pearson correlation for the growth zone variables.

		Main decider for his life	Preference toward experiences leading to personal growth	Fulfilling dreams as a tourist	Seeking experiences leading to emotional growth	Travel as lifestyle
Main decider for his life	Pearson Correlation (r)	1				
	Sig. (2-tailed)					
Preference toward experiences leading to personal growth	Pearson Correlation (r)	0.484	1			
	Sig. (2-tailed)	0.000				
Fulfilling dreams as a tourist	Pearson Correlation (r)	0.412	0.374	1		
	Sig. (2-tailed)	0.000	0.000			
Seeking experiences leading to emotional growth	Pearson Correlation (r)	0.323	0.484	0.435	1	
	Sig. (2-tailed)	0.000	0.000	0.001		
Travel as lifestyle	Pearson Correlation (r)	0.172	0.318	0.368	0.493	1
	Sig. (2-tailed)	0.000	0.000	0.002	0.000	

Source: own computation.

Although all correlations among variable attributes of the growth zone are significant (Sig = 0.000 < 0.05) and positive (r correlation > 0), we noticed no strong or very strong correlations. Most of the correlations are weak, where the r-correlation is situated between 0.2 and 0.4. We found several moderate correlations (*r* = 0.4–0.6) which could be further discussed:

The correlation between “Travel as lifestyle” and “Seeking experiences leading to emotional growth,” *r* = 0.493.

The correlation between “Preference toward experiences leading to personal growth: and “Main decider for his life,” *r* = 0.484.

The correlation between “Preference toward experiences leading to personal growth” and “Seeking experiences leading to emotional growth,” *r* = 0.484.

The correlation between “Main decider for his life” and “Fulfilling dreams as a tourist,” *r* = 0.412.

The correlation between “Fulfilling dreams as a tourist” and “Seeking experiences leading to emotional growth,” *r* = 0.435.

We used the Cronbach’s Alpha coefficient of reliability to identify the measure of internal consistency among the items defining the growth zone, calculated by SPSS software. The aim was to check whether the items contribute to the comfort zone significance or not. The value of Cronbach’s Alpha was 0.748 which indicated an acceptable consistency (Tavakol and Dennick, 2011). This consistency could not be improved through the removal of any item.

As a conclusion of this test, we can state that the growth zone items do have an acceptable consistency which means there is consistency among the answers given by respondents for this dimension. This will lead to identifying the specific behaviors of tourists choosing a certain destination from the growth zone.

## Discussion

Senninger’s Learning Model is an evergreen one and, moreover, is proving to be a transversal one. It explains the foundations of decision making process. All the time the human mind tries to arbitrate between staying safe and daring for more, between remaining in the comfort zone and overcoming fear of leaving it. The comfort zone is, no matter what, the reference system of all the other levels, even if you decide to buy bread or an electric car (Wallace and Lãzãroiu, 2021; Popescu et al., 2022), to choose between staying home or discover a new destination (Andronie et al., 2021; Nica, 2021; Pop et al., 2022; Robinson, 2022).

In tourism and travel, various companies or even cities understood that a traveling decision is facing two alternatives: (1) not to change a thing and repeat a previous choice (like staying home or choosing all over again the same tested destination) and (2) pointing out new destinations, new experiences, new adventures (Pop et al., 2022). So a question is rising: the tourist offer must include arguments for both types of travelers or must be a focused one? Spontaneously we might think dichotomicly: you must be either unique or you do not count. But the reality shows that we can have smart cities, with a smart infrastructure and integrating IoT, but offering also traditional well conserved historical areas (Andronie et al., 2021; Nica, 2021; Robinson, 2022). Some will come for tasting new experiences and some will be attracted by nostalgic reasons.

In the end everything is a segmentation issue. For different targets you must have different arguments. That is why our research can be a basis for including a new criterion to the segmentation strategy for tourist products and services. By knowing what particular learning zone is the most important in making a travel decision for a certain segment of clients a company can adapt the offer. The case of Gen Z consumers is particularly interesting, because they are the future most important travelers. They are highly educated, social and environmental activists, digital natives and, extremely important, the most significant buyers all over the globe. They know to find without any help the most reliable information online (Popescu Ljungholm, 2022), they are present on various social media and are the most probably to leave a review. In the light of our research model we can ask ourselves: should we treat Gen Z equally, like we used to do with all the other generations before (decision made from our comfort zone)? Should we fear them and decide that they are beyond our marketing possibilities (decision made from our fear zone)? Should we try to understand them (decision made from our learning zone)? Or should we decide to grow with them (Popescu Ljungholm, 2022), to thrive together (decision made from our growth zone)?

Our research offers a glimpse into a very actual and important question: is the buying decision impacted by one of these four learning zones? We added a new perspective to the well-known Senninger’s model, one referring to choosing the next travel destination. We have experienced the Covid-19 pandemic situation and tourism and travel sector was one of the most affected ones. We hesitated to travel because of fear. We chosed to stay safe and we remained home for years. Now, in 2022, we are facing the same old decision related to travel destinations. What we have noticed is that Gen Z dare to exit their comfort zone and to go beyond fear, driven by learning and growth reasons. We still do not know how responded other generations or if Gen Z have the same response for every decisions, no matter the domain.

The main contribution is that we can offer a measuring scale for the 4 zones of the Learning Zone Model. The particularity lies in applying this model to tourism. It opens new possibilities for the model to be applied to other fields as well alongside new possibilities for statistical determinants through inferential statistics. Moreover, understanding the zone where a decision is made, choosing a destination or other products or services allows us to profile better the consumer from a psychological perspective.

## Conclusion

The present research explored the Gen Z tourist’s decision for their next holiday. As a theoretical implication, we started by creating a scale based on the 4 zones corresponding to Senninger’s model. Our scale had 20 items (5 statements for each zone/level of the model) regarding choosing the next travel destination and it is measured from 1 to 10 according to the extent to which a respondent agrees, where 1 is full disagreement and 10 is full agreement (Likert scale). Each section involved one key statement which contains the name of the interest zone (e.g., comfort zone).

As a future research perspective, our intention for this statements is to be used in further inferential statistics as part of future research. This key statement had scored consistently the best evaluation as per Cronbach’s Alpha test.

The managerial implications can be helped by our findings. We consider that the Senninger’s Learning Model can provide segmentation criteria (comfort seekers/fear dominants/learners and thrivers) for a new variable: learning type.

To support that, we say that all the statements were based on collected data from tourism specialists. They describe the tourists choosing a travel destination from within their comfort zone as being focused on control and security, being persons who try to mitigate any risks. Therefore they choose their travel destinations depending on security and lack of unforeseen situations criteria. They also rely on repeating positive experiences. Our quantitative research shows for the comfort zone the strongest correlation is between the willingness to mitigate risks and uncertainty avoidance which could take this type of tourist out of his comfort zone. A second strong correlation found was between security-inspired choices and uncertainty avoidance.

According to the specialists choosing a certain travel destination from within the fear zone can be mostly explained through a high degree of being suggestible but also curious and making efforts to overcome the fear of the unknown, lack of excessive planning and welcoming of new challenges. We used those descriptors in realizing our survey and we found out the strongest correlation among the variables of the fear zone was in fact a moderate one. It was the correlation between lack of excessive planning and accepting new challenges. A second reasonable correlation was between new experiences and overcoming the fear of the unknown. All the other correlations within the fear zone were weak toward moderate.

Travelers choosing their destination from within the learning zone were depicted by the specialists as being open to novelty, curious, eager to learn, adventurous, and accepting challenges as well as risks. Our survey results indicated that the Learning zone is the most relevant for Gen Z tourists from Iasi when choosing a travel destination. We recorded the strongest correlations here such as between being adventurous and opened to new experiences; being adventurous and accepting new challenges; being opened to new experiences and a preference for challenges; being adventurous and learning new things and embracing new challenges and the readiness to learn new things. The other correlations were moderate toward good.

For the travelers in the growth zone, the destination choice involves fulfilling certain ideals and objectives from a touristic point of view. Experiences that involve emotional development, passion, decisiveness, personal growth, accepting risks, and perceiving travel as a lifestyle are the most important for them. While most of the correlations are weak, we found, however, a few reasonable correlations: (i) between travel as a lifestyle and seeking experiences leading to emotional growth, (ii) between inclination toward experiences leading to personal development and decisiveness, (iii) between seeking experiences leading to emotional growth and inclination toward experiences leading to personal development, (iv) between decisiveness and fulfilling dreams as a tourist, and (v) between seeking experiences.

To sum up, we can state that the Gen Z tourist from Iasi displays behaviors that can be associated with learning or growth zones rather than the comfort zone. This is relevant when choosing the next travel destination.

As limitations, we can mention that the sample was limited to 209 individuals, a number relatively small to be statistically representative for the Gen Z population of Iasi. The sample’s structure is heterogenic, having more female respondents. We operated with a convenience non-probability sample.

At the theoretical level the model used as the fundament of this research is the Learning Zone Model (Senninger, 2000) which consists of the 4 zones (comfort, fear, learning, and growth) do not offer a clear differentiation of those zones. We cannot assign a precise zone to each tourist since the model was conceived as more of a progressive path.

The research method, an online survey, might reflect the main reason for the lack of representativity of the sample. Since the survey was distributed online using various social media platforms, there was a lack of control over the respondents. Moreover, the collection of data was carried out during the last phase of COVID-19 pandemic restrictions which involved a relevant transition from online to offline.

## Data availability statement

The original contributions presented in this study are included in the article/supplementary material, further inquiries can be directed to the corresponding author.

## Ethics statement

The studies involving human participants were reviewed and approved by Faculty of Economics and Business Administration, at University Alexandru Ioan Cuza of Iasi. Written informed consent for participation was not required for this study in accordance with the national legislation and the institutional requirements.

## Author contributions

All authors listed have made a substantial, direct, and intellectual contribution to the work, and approved it for publication.
